# Binocular vision supports the development of scene segmentation capabilities: Evidence from a deep learning model

**DOI:** 10.1167/jov.21.7.13

**Published:** 2021-07-21

**Authors:** Ross Goutcher, Christian Barrington, Paul B. Hibbard, Bruce Graham

**Affiliations:** 1 Psychology Division, Faculty of Natural Sciences, University of Stirling, Stirling, UK; 2 Psychology Division, Faculty of Natural Sciences, University of Stirling, Stirling, UK; 3 Computing Science and Mathematics Division, Faculty of Natural Sciences, University of Stirling, Stirling, UK; 4 Department of Psychology, University of Essex, Colchester, UK; 5 Computing Science and Mathematics Division, Faculty of Natural Sciences, University of Stirling, Stirling, UK

**Keywords:** deep learning, binocular vision, segmentation, depth perception

## Abstract

The application of deep learning techniques has led to substantial progress in solving a number of critical problems in machine vision, including fundamental problems of scene segmentation and depth estimation. Here, we report a novel deep neural network model, capable of simultaneous scene segmentation and depth estimation from a pair of binocular images. By manipulating the arrangement of binocular image pairs, presenting the model with standard left-right image pairs, identical image pairs or swapped left-right images, we show that performance levels depend on the presence of appropriate binocular image arrangements. Segmentation and depth estimation performance are both impaired when images are swapped. Segmentation performance levels are maintained, however, for identical image pairs, despite the absence of binocular disparity information. Critically, these performance levels exceed those found for an equivalent, monocularly trained, segmentation model. These results provide evidence that binocular image differences support both the direct recovery of depth and segmentation information, and the enhanced learning of monocular segmentation signals. This finding suggests that binocular vision may play an important role in visual development. Better understanding of this role may hold implications for the study and treatment of developmentally acquired perceptual impairments.

## Introduction

In both biological and artificial systems, visual processing supports the recovery of critical environmental properties, such as segmentation of figure from background, and the localization of objects in depth. In biological systems, numerous visual cues have been identified as supporting both figure-ground segmentation (e.g., Wagemans, Elder, Kubovy, Palmer, Peterson, Singh, & von der Heydt,2012) and the measurement of depth (e.g., Cutting & Vishton, 1995; Howard & Rogers, 2012; Welchman, 2016). These cues have typically formed the basis of efforts to model the processing of depth and object segmentation in the brain (e.g., Elder, Krupnik, & Johnston, 2003; Elder, 2018; Hildreth & Royden, 2011; Langer, Zheng, & Rezvankhah, 2016; Watt, Ledgeway, & Dakin, 2008). In machine vision research, problems of scene segmentation and depth estimation have been similarly addressed to support multiple applications including the guidance of autonomous vehicles (e.g., Smolyanskiy, Kamenev, & Birchfield, 2018), object tracking (Wang, Zhang, Bertinetto, Hu, & Torr, 2019) and enhanced scene understanding (e.g., Garcia-Garcia, Orts-Escolano, Oprea, Villena-Martinez, & Garcia-Rodriguez, 2017; Huang, Matzen, Kopf, Ahuja, & Huang, 2018; Jégou, Drozdzal, Vazquez, Romero, & Bengio, 2017; Song, Lichtenberg, & Xiao, 2015; Wang & Shen, 2018).

Despite these overlapping concerns, substantial differences exist between the modeling approaches used in biological vision research, and those used in the development of machine vision systems. Despite notable early exceptions (e.g., Marr & Hildreth, 1980; Marr & Poggio, 1979), models of biological visual processing have often focused on accounting for the performance of human observers on an array of psychophysical tasks (e.g., Banks, Gepshtein, & Landy, 2004; Goutcher, 2016; Henricksen, Cumming, & Read, 2016; Hillis, Ernst, Banks, & Landy, 2002; Lovell, Bloj, & Harris, 2012). Many such models also constrain themselves to consider processing in biologically plausible terms, often focusing on the combination of physiologically inspired receptive field structures or other established response properties of neurons or neuronal populations in visual cortex (e.g., Banks et al., 2004; Chauhan, Masquelier, Montlibert, & Cottereau, 2018; Ecke, Papp, & Mallot, 2021; Goncalves & Welchman, 2017; Henricksen et al., 2016; Maiello, Chessa, Bex, & Scolari, 2020; May, Zhaoping, & Hibbard, 2012; Watt et al., 2008). Indeed, for a subset of physiologically inspired models, a primary aim is to account for the functioning or organization of particular sets of neurons, rather than to directly solve how such neurons contribute to specific visual or visuomotor behaviors (e.g., Bredfeldt, Read, & Cumming, 2009; DeAngelis, Ohzawa, & Freeman, 1991).

In contrast, machine vision research has largely focused on providing exactly these solutions. As such, the goal for machine vision models has been to maximize the precision and accuracy of performance, typically in estimation and categorization tasks, with real world, or close to real world, scenes. This typically means that such models disregard some of the peculiarities of biological visual processing that are often highlighted in the use of specific stimuli and experimental designs (e.g., Goutcher, Connelly & Hibbard, 2018; Goutcher & Wilcox, 2016; Goutcher & Wilcox, 2021; Harris, 2014; Kingdom, Yared, Hibbard, & May, 2020; Wardle, Palmisano, & Gillam, 2014; Wardle & Gillam, 2016). Yet these more targeted examinations of specific effects in visual processing (e.g., depth illusions, bias, etc.) can often reveal critical information about visual signals and visual processing that might otherwise be overlooked in the search for solutions to real world tasks. In this article, we show how a consideration of the visual cues for scene segmentation and depth measurement may inform the development of a deep neural network (DNN) model of these processes. This model was focused on examining the efficacy of binocular cues, which we consider in detail below.

### Binocular signals for depth estimation and scene segmentation

Depth estimation and scene segmentation are two fundamental problems for visual processing. Depth estimation problems include the measurement of egocentric distance, as well as the measurement of relative depth and the estimation of three-dimensional (3D) object shape (cf., Parker, 2007; Howard & Rogers, 2012; Welchman, 2016). Scene segmentation problems can be similarly subdivided, delineating problems of figure-ground segmentation, object boundary classification, semantic segmentation and instance segmentation (Garcia-Garcia et al., 2017; Long, Shelhamer, & Darrell, 2015). In this article, we consider the problems of estimating egocentric distance, defined as the normalized absolute distance to the observer for each pixel in a scene, and semantic segmentation, defined as the production of a pixel-by-pixel map defining the identity and location of each object in a scene.

For depth estimation, numerous visual cues have been identified as informative of depth structure, including pictorial cues, such as shape-from-shading, texture gradients, and linear perspective, and dynamic cues such as motion parallax and structure-from-motion (cf., Howard & Rogers, 2012; Welchman, 2016). A similar array of cues has been proposed for scene segmentation, including the identification of edges defined by differences in luminance, contrast, color, texture and/or motion (e.g. Martin, Fowlkes, & Malik, 2004; Sundberg, Brox, Maire, Arbeláez, & Malik, 2011), as well as principles for the grouping and interpretation of such edges (Wagemans et al., 2012).

Binocular images provide a particularly important source of information for both depth estimation and scene segmentation. The role of binocular signals for depth estimation is relatively uncontroversial; small positional differences between left and right eye images, known as binocular disparities, are highly informative of the 3D structure of the distal scene. Use of this depth cue depends on the resolution of the problem of binocular correspondence, where matching points are found between left and right eye images. Numerous rules, constraints and heuristics have been proposed for correspondence resolution in biological vision (e.g., Goutcher & Hibbard, 2010; Goutcher & Hibbard, 2014; Marr & Poggio, 1979), many of which are implicitly implemented in biologically inspired algorithms that measure binocular disparity through cross-correlation, or cross-correlation-like processes (Banks et al, 2004; Qian & Zhu, 1997; Read & Cumming, 2007). Such models are often derived from the binocular energy model, where the energy at a given disparity is measured as the sum of quadrature pairs of phase or position-shifted binocular simple cell receptive fields (DeAngelis et al, 1991; Fleet, Wagner & Heeger 1996). The use of such constraints is also evident in the measurement and optimization processes of many classical machine vision models of depth estimation (Hirschmulller, 2005; Scharstein & Szeliski, 2002).

In addition to their role in depth estimation, binocular images also contain important signals for scene segmentation. Depth differences at object boundaries may give rise to areas of binocular half-occlusion, where regions of an image are visible to one eye only (Harris & Wilcox, 2009; Nakayama & Shimojo, 1990; Tsirlin, Wilcox, & Allison, 2010). This absence of matching regions between left and right eyes results in changing patterns of disparity energy, where unmatched regions are likely to be associated with generally low levels of binocular correlation across a range of potential disparity values, and where the local image structure at these unmatched regions is more likely to match neighboring “background” image areas (Basgöze, White, Burge, & Cooper, 2020). Where binocular disparity information is available, disparity-defined boundaries are typically associated with large disparity gradients (Basgöze et al., 2020; Cammack & Harris, 2016; Goutcher et al., 2018; Goutcher & Wilcox, 2021). Note that, for both half-occlusion and disparity-defined boundaries, binocular segmentation signals are the result of image-based, rather than depth or distance-based, computations. This contrasts with many machine vision models of segmentation, where monocular images may be supplemented by an explicit depth channel, rather than make direct use of binocular imaging (Eitel, Springenberg, Spinello, Riedmiller, & Burgard, 2015; Silberman, Hoiem, Kohli, & Fergus, 2012; Song et al., 2015, although see some early work by, for example, Birchfield & Tomasi, 1999). The DNN model detailed in this article makes use of binocular image inputs to provide access to these image-based cues.

### Deep learning as a tool for understanding the brain

Given the, often diverging, purposes of modeling endeavors in biological and machine vision research, one may wonder whether there are any benefits to utilizing machine vision approaches in an attempt to understand biological systems. Recently, this question has come under renewed focus with the rise of deep learning approaches in machine vision (cf., Lopez-Rubio, 2018; Majaj & Pelli, 2018; Richards et al., 2019). For many researchers in biological vision, deep learning networks provide an attractive and powerful way to conceive of the processes occurring in the mammalian visual system (Kriegeskorte, 2015; Rideaux & Welchman, 2020; Rideaux & Welchman, 2021; Srinath, Emonds, Wang, Lempel, Dunn-Weiss, Connor, & Nielsen, 2020). Like cells in the visual pathway, from retina to cortex, the filtering operations in DNNs make use of operations such as convolutions and max pooling, with some model architectures (e.g., “AlexNet”; Krizhevsky, Sutskever, & Hinton, 2017) exhibiting filter weights that bear similarity to the excitatory-inhibitory receptive field structures found in retinal ganglion cells, LGN and primary visual cortex. The activity of these forms of DNN has been used to draw inferences about the processing potential of areas further along the visuo-cortical pathways (e.g., Srinath et al., 2020). Yet many of the model architectures used in machine vision differ significantly from the processing pathways seen in biological visual systems. The development of DNN models also typically depends on supervised learning processes that differ markedly from the kinds of feedback available to active organisms (for detailed discussion, see Majaj & Pelli, 2018). Together, these differences suggest that, at the very least, substantial care must be taken when drawing comparisons between the activity in DNNs and the processing occurring in biological systems.

There is, however, another way to make use of DNN performance as a tool for understanding biological vision. Rather than consider DNNs as intrinsically informative of the processes occurring in biological visual systems, one may instead consider such networks as informative of the signals present in the input images. Thus, one may consider the capacity of DNNs to successfully perform a given task (e.g., segmentation and/or depth estimation) as indicative of the presence of task-relevant information within the input images and of its encoding by the network. By extension, one may therefore consider changes in performance in response to a principled stimulus manipulation as indicative of the efficacy of the manipulated stimulus information for the model's set task.

This approach is similar to existing model-based analysis methods, such as Bayesian-derived ideal observer-based measures of efficiency (Barlow, 1962; Pelli, 1990; Pelli, & Farell, 1999) and the application of support vector machines, for example in the classification of signal-relevant responses in neuroimaging data (LaConte, Strother, Cherkassky, Anderson, & Hu, 2005). It also extends image analysis-based approaches aimed at understanding the statistics of natural scenes (e.g., Adams, Elder, Graf, & Muryy, 2016; Burge & Geisler, 2014; Fowlkes, Martin, & Malik, 2007; Hunter & Hibbard, 2018) by determining whether useful visual information is readily recoverable. Using this approach, one may distinguish between signals that are *in principle* informative for a given task, and those that, given the complex, multi-object structures found in natural scenes, a sensory system can actually learn to use effectively. This can be done by focusing on the input sensory information, and output task performance, without there necessarily being any direct relationship between the properties of the DNN hidden layers, and any particular features of the biological visual system (López-Rubio, 2018). Under this approach, network architectures can be considered as hypotheses on the importance of specific image properties for the task(s) under investigation. In this article, we examine the learning of depth estimation and segmentation signals from binocularly presented, rendered, multi-object scenes and examine the role played by binocular signals in this process. We show that manipulation of binocular image signals significantly impacts on model performance in both depth estimation and scene segmentation tasks, highlighting the importance of binocular viewing for these tasks. We further show that inputs from scene segmentation pathways in our network significantly enhance depth estimation performance.

## Methods

To assess the importance of binocular cues for scene segmentation and depth estimation, we developed a DNN that took binocular images as inputs. This model was structured as an encoder-decoder network, an architecture that has previously proven useful for both segmentation and depth estimation tasks (Garcia-Garcia et al., 2017; Wang & Shen, 2018). Specifically, our architecture was based on the U-net network developed by Ronneberger, Fischer and Brox (2015). Following initial layers of feature extraction, left and right image input pathways converged on a common binocular stage. Subsequent processing stages were separated into parallel pathways for segmentation and depth estimation, producing a single set of depth estimates for distance to the left camera, and object identity segmentation maps for both left and right camera images. The model was trained on both tasks simultaneously.

### A 3D rendered image training set

**Figure 1.**
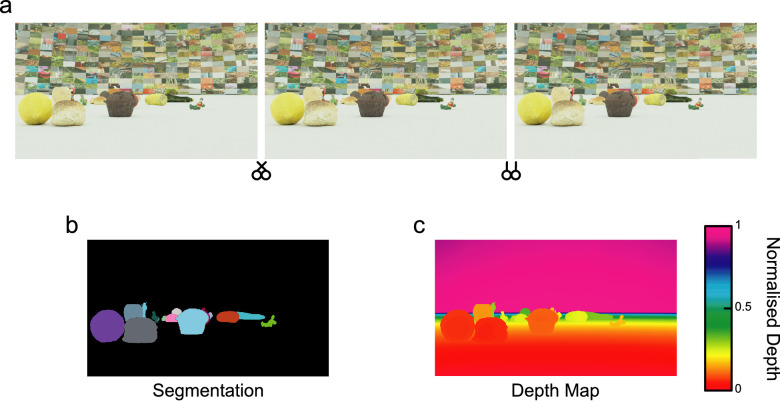
Example images from the dataset (a) arranged for crossed and uncrossed free fusion, together with (b) segmentation and (c) normalized depth ground truths. Segmentation image colors label each object category (see Figure 4b for details). Normalized depth values vary from 0 (closest, in red) to 1 (farthest, in violet).

We constructed a training dataset of complex scenes, each containing multiple objects. Scenes were constructed using Blender (Blender Foundation, Amsterdam, Netherlands), and objects were drawn from an existing dataset of high-quality 3D renders of real objects (Solid Sight Dataset – Hibbard, Scarfe, Hornsey, & Hunter, 2016). Objects were scanned using a NextEngine 3D laser scanner (NextEngine Inc., Santa Monica, CA, USA), creating high-density 3D models of the object. Our complex scenes each contained 24 distinct objects from the dataset, arranged in pseudo-random positions to mimic objects distributed across a circular flat surface of radius 5.2 m. Scanned objects were an array of fruits, vegetables and toys, rendered and captured under a diffuse light source. Each scene was viewed within a hemispherical domed “sky” covering the full circular area of the scene. This was textured using image samples taken from the McGill Calibrated Colour Image Dataset (Olmos & Kingdom, 2004). Image samples were used to ensure that segmentation was not overly simplified by the presence of large blank areas, but instead were comprised of image information consistent with the statistics of natural scenes. Example images and ground truths are shown in Figure 1.

To train our networks we generated 40 such scenes, capturing pairs of 224 × 224 pixel RGB images from laterally separated binocular cameras. Images were captured for 50 frames following pseudo-random walks around the perimeter of the scene. The inter-camera distance was 6.5 cm, with parallel viewing geometry. Training images were accompanied by pixel-by-pixel ground-truth segmentation images for the left and right camera and equivalent depth maps, obtained as part of the image rendering process. Segmentation maps used One-Hot encoding (cf., Cerda, Varoquaux, & Kégl, 2018) to specify categories, matched to a colourmap for later visualization. Depth map values were normalized to fall between values of 0 and 1, with 0 being the value closest to the camera and 1 being the farthest value. Depth values were therefore on a relative scale, although, in practice, nearest and farthest distances were equivalent between scenes. In addition to this initial training set, we generated a further validation set of seven scenes, containing pairs of images for 50 frames to check trained model performance. All results reported below used a final test set of four novel scenes not previously presented to the model. The use of identical test images allowed for statistical analysis using related-samples approaches.

### Model architecture and training regime

**Figure 2.**
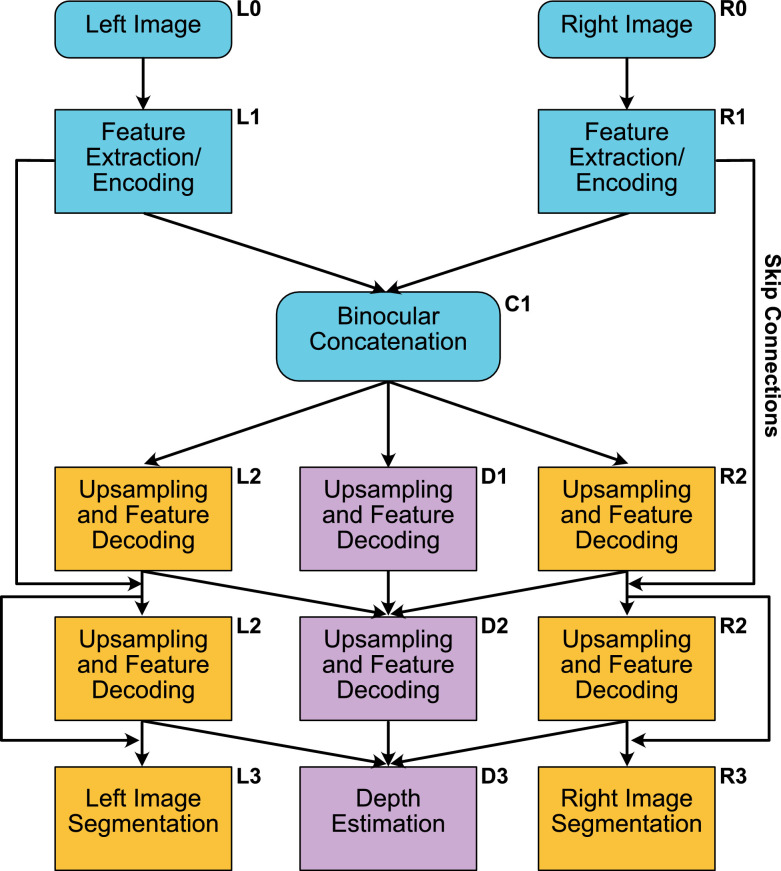
Illustration of the network architecture. Left and right input pathways feed into a common binocular concatenation stage, leading to distinct segmentation and depth estimation output pathways. Full details of the operations at each network layer are provided in Supplementary Table S1.

Our binocular encoder-decoder model is built on a TensorFlow 2.x and Keras backbone. In this model, features are encoded from the RGB image pair inputs and fed into a common binocular stage for the simultaneous learning of segmentation and depth from a shared feature pool. The binocular stage is a concatenation of prior monocular filter pathways, allowing differences in filter structure at equivalent image locations to affect subsequent processing. Information from this binocular concatenation stage is fed forward into the three output branches, left image segmentation, right image segmentation and depth prediction. As stated above, depth is calculated as normalized distance. This was calculated relative to the left camera only. An illustration of the model architecture is shown in Figure 2, with full details provided in Supplementary Table S1. There were a total of 10,901,923 parameters in the network, with 10,889,955 trainable parameters and 11,968 nontrainable parameters. Code for the network is available at the following URL: https://github.com/StirlingChris/User_Version/tree/master.

The model consisted of five types of layers: convolutional, batch normalization, maximum pooling, up-sampling, SoftMax and skip connections/residual units. We used a convolutional filter size of 3 × 3 and a stride of one for all convolutional layers. Batch normalization is used to rescale the values of the results between zero and one to improve model efficiency and stability. We also utilized maximum pooling to summarize and reduce the dimensionality of the extracted features, decreasing the number of parameters in the overall model and the size of the input into the next layer. The up-sampling modules are used when decoding the features extracted and to increase the size of the input so that the output is the same size as the ground-truths the model is trained on. Skip connections have been shown to improve the passing on of information between layers and to help preserve spatial information (He, Zhang, Ren, & Sun, 2016; Jégou et al., 2017), which is of particular use for our model. We used skip connections to pass information between modules of matching size in the encoding and decoding ends of the network.

The output layer of the segmentation branch consists of SoftMax neurons, which output a probability that each pixel is a given class, producing a 224 × 224 RGB segmentation map as output. The argmax of all the SoftMax outputs is then taken as the most likely class for each pixel. The number of SoftMax neurons determines the maximum number of potential outputs. As such, we used 25 SoftMax neurons; one for each possible object category (24 object categories, plus background) in our dataset.

An additional feature of our model is that we introduced connections between these segmentation pathways and the depth branch. The depth branch of the network has features from both segmentation branches fed into it at 3 dimensionalities: 28 × 28, 56 × 56 and 112 × 112. These features are then fed forward to the linear activation output unit to produce a depth map of a 224 × 244 × 1 image as output. Note that there were, however, no direct connections from the network's depth branch to the segmentation branches. This means that any depth-based segmentation cues must be derived directly from underlying image properties and cannot be due to the explicit measurement of depth. The potential benefit of these connections instead lies in the enhancement of depth estimation processes. Several recent psychophysical findings (Cammack & Harris, 2016; Deas & Wilcox, 2014, 2015; Goutcher et al. 2018; Goutcher & Wilcox, 2021; Mamassian & Zannoli, 2020) point to the importance of segmentation boundaries in the quantitative perception of binocular depth. By explicitly manipulating the input of segmentation information into depth measurement processes, we tested whether our network was able to learn to make use of such signals.

The network was trained using the 40 binocular training scenes described in section 2.1, where each scene contained 50 binocular, 224 × 224 pixel, RGB frames. No data augmentation was used during our training processes, except for vertical flips which were tested but offered little for improving performance while also increasing training time. Standard data augmentation procedures were avoided, as these alter available disparity signals and would, therefore, affect the learning of disparity dependent signals. No dropout was used on any part of the network.

The model was trained using the Adam optimizer with learning rate α = 0.001, β_1_ = 0.9, β_2_ = 0.999, ε = 1e -07, where α controls the step-size for weight changes on each iteration, β_1_ and β_2_ control the decay rate on moving averages of the first and second moment of the estimated gradient, and ε prevents division by zero (see Kingma & Ba, 2014, for further details). The ReLu activation function was used on all units except for the output neurons, which used SoftMax and a linear activation. We trained for a maximum of 150 epochs, using a batch size of 8 image pairs and a step size of 32. Image pairs were shuffled between epochs. Model performance was calculated on each iteration using a categorical cross-entry loss function for each segmentation pathway and by finding the per image root mean squared error (RMSE) for the depth estimation pathway. The total loss for the model was taken as the sum of these measures. An early-stopping condition was specified, where training was stopped and “best performing” weights saved if performance failed to improve for 15 consecutive epochs. Best performance was measured as minimized validation loss. Model weights at each layer of the network were held constant following the completion of these training regimes.

### Binocular image manipulation

To examine the contribution of binocular image signals to model performance, we tested our DNN under three distinct viewing conditions. Following training and validation, the model was presented with test images under standard viewing conditions, identical to the image arrangements used in training, identical images or images swapped between left and right cameras. This type of swapped image presentation is typically referred to as *pseudoscopic viewing*, after the device developed by Charles Wheatstone (1852). The presentation of identical images removes all binocular disparity and monocular occlusion cues from the input images, while swapped presentation reverses these signals while leaving monocular depth and segmentation cues intact. All image manipulations were accompanied by appropriate adjustments to ground truth data, where these data were tied directly to the presented image(s). Thus, for pseudoscopic viewing, ground truth data was in direct opposition to binocular disparity-defined depth.

As a further examination of the contribution of binocular image signals, we also trained and tested adapted versions of our network. First, we ran a fully monocular version of our model, based on the U-net architecture (Ronneberger et al., 2015) from which our binocular network is derived. Our U-net architecture focused on segmentation only and used only single images as inputs. There were a total of 3,120,921 parameters in the U-net model, with 3,117,049 trainable parameters and 3,872 nontrainable parameters. The reduction in parameters compared with our binocular network is due to the absence of the depth estimation pathway and the reduction to only a single image input and single segmentation output pathway. The models, and number of equivalent parameters, are identical in all other respects. As a further comparison with the monocular U-net model, we also trained a segmentation only version of our binocular model. This version of our network was trained on binocular images, as with our standard approach, but with learning guided only by the segmentation loss functions.

In addition to training the monocular U-net model and segmentation only version of our binocular model, we also trained and tested our binocular model with identical inputs only. This variation on our approach allowed for a direct comparison of the effects of binocular image presentation within the same model architecture. Further comparison with the results of the U-net model additionally allowed for an assessment of the benefits of binocular viewing even in the absence of binocular disparity signals, for example, through binocular summation (Baker, Lygo, Meese, & Georgeson, 2018; Blake & Fox, 1973; Blake, Sloane, & Fox, 1981).

## Results

**Figure 3.**
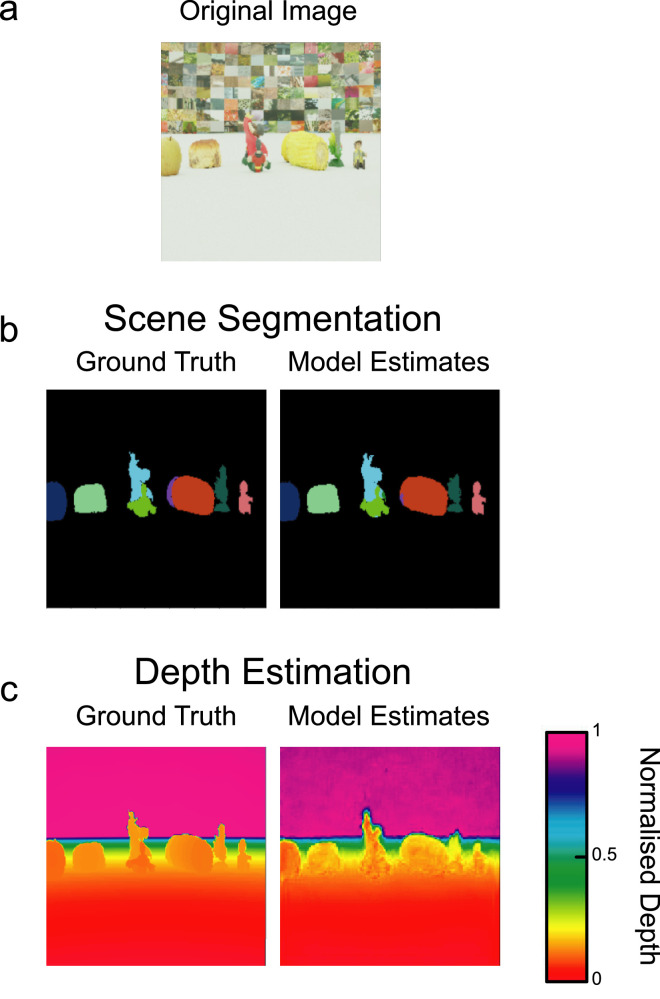
Model outputs for an example image (a), provided with comparison to ground truth images for (b) scene segmentation and (c) depth estimation. Outputs, images, and ground truths are shown for the left image only, because this was the basis for all depth calculations.

Figure 3 shows example segmentation and depth estimation outputs from the binocular image trained network, alongside ground truth images. Segmentation performance, including object identification, was highly accurate for binocular images, as was the estimation of depth at each image location. Simultaneous segmentation and depth estimates were also produced in near real-time, providing output images in a processing time of 136 ms (∼8 frames on a 60Hz display). Timing estimates were obtained from a machine running the network with a GeForce GTX 1080 GPU.

### Depth estimation performance

To quantify model performance for standard binocular image presentation, depth estimation errors were taken as the difference between estimated and ground truth depth values for each point in the image. Errors could vary between ±1, with negative errors indicating over-estimation of relative depth, and positive values indicating under-estimation. Depth errors were calculated on a per-pixel basis, for all images to provide measures of the distribution of depth errors across the test image set. Mean depth errors were 0.016, with a standard deviation of 0.052, indicating a slight bias for positive (i.e., underestimation) errors. As a further summary of depth errors, we also calculated the average unsigned error (RMSE), which was 0.053 across all images, with a standard deviation of 0.013.

### Scene segmentation performance

**Figure 4.**
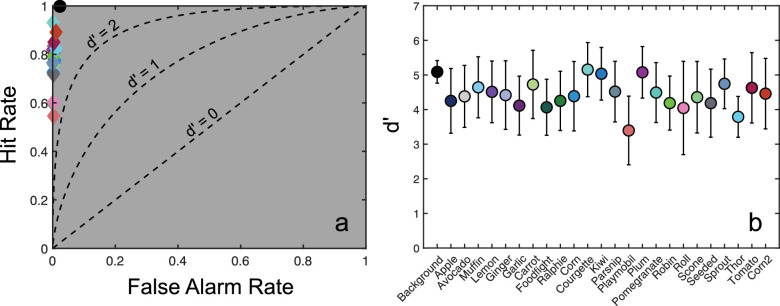
(a) Model segmentation performance for each object, plotted as the Hit rate against the Object False Alarm rate. Better segmentation performance is indicated by points lying closer to the top-left corner of this graph. Curves plotting differing levels of performance as dʹ scores are shown for comparison. (b) Average dʹ scores for each object in the dataset. *Error bars* show standard deviations, colors are matched to datapoints shown in (a).

Pixel-by-pixel segmentation accuracy averaged 98.8% across all 100 test images (min 89.6%, max 99.8%). To further quantify the model's segmentation performance under standard binocular image presentation, we calculated the proportion of pixels on each image correctly identified as belonging to each object class, plus a “background” class (the Hit rate). In addition, we calculated the proportion of pixels mistakenly identified as belonging to each class (the False Alarm rate). There are multiple potential metrics for calculating these False Alarm rates. One may consider the proportion of pixels misidentified as belonging to the target object, either for all non-target pixels (including background pixels), or for non-target pixels that belong to another object (i.e., non-target, non-background pixels). We used this latter, more conservative, measure in all segmentation analyses. We refer to this as the Object False Alarm rate. These results are plotted in Figure 4a.

Segmentation hit rates were typically high (averaging 79% across objects), with consistently very low object false alarm rates, averaging 0.3%. False alarm rates were highest for the background pixels, indicating that target pixels were typically misidentified as belonging to the background. These values were summarized as dʹ scores and are plotted for each object in Figure 4b. Average dʹ values of 4.4 were found across objects and images. This value can be understood as quantifying the strength of the classification decision information available to the model, relative to the standard deviation of the noise in these decisions.

### Quantifying the contribution of binocular signals

**Figure 5.**
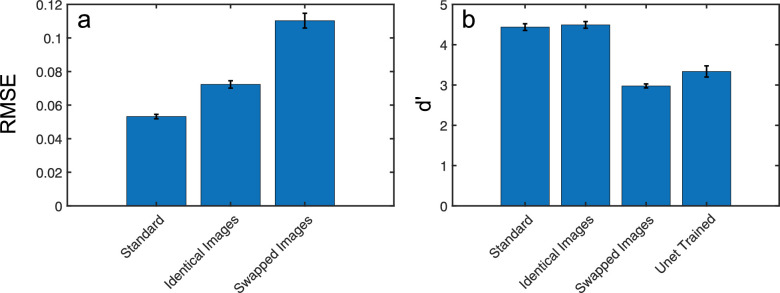
(a) Depth errors (RMSE) for binocular presentation, identical images, and swapped images. Errors increase for non-standard binocular presentation. (b) Segmentation performance plotted as dʹ scores across all objects for the same presentation arrangements as in (a), plus the monocular U-net segmentation model. Reductions in dʹ scores show poorer segmentation performance for swapped and U-net trained models, compared to standard binocular presentation. *Error bars* show standard errors on the mean.

The use of different binocular viewing conditions allows for a more detailed understanding of the contributions of binocular signals to depth estimation and scene segmentation performance. We presented our model with correctly arranged left-right images, identical images or left-right swapped, pseudoscopic images. These latter manipulations have the effects of, respectively, removing or reversing binocular depth signals. Mean depth errors and segmentation performance for these models are shown in Figure 5. We also compared segmentation performance to a version of the monocular U-net model (Ronneberger et al., 2015), trained on our dataset. As noted in section 2.3, the segmentation pathway for our model used equivalent processing stages to the U-net model, except for the presence of the binocular convergence stage. As such, it offers a clear comparison for the benefits of binocular presentation for both learning and testing.

Depth estimation errors (Figure 5a) increased from average RMSE values of 0.053 across all 100 test images for the standard model, to 0.072 and 0.110 for identical image and swapped image conditions, respectively. These differences were significant on Bonferroni corrected, two-tailed, related-samples t-tests (*t_99_* = 14.97, *p* < 0.001, Cohen's *d* = 1.5; *t_99_* = 16.78, *p* < 0.001, Cohen's *d* = 2.0; *t_99_* = 11.44, *p* < 0.001, Cohen's *d* = 1.4, for standard-same, standard-swapped, and same-swapped comparisons, respectively). These results indicate that our model is able to use monocular, pictorial depth cues, while also showing sensitivity to eye-of-origin dependent binocular image differences.

The segmentation pathway shows a similar pattern of results (Figure 5b): dʹ values were 4.44 for the standard model, 4.49 for identical images and 2.98 for swapped images. Analysis with related-sample *t*-tests, using mean dʹ values for each object showed significant differences between standard and swapped presentation (*t*_24_ = 17.05, *p* < 0.001, Cohen's *d* = 3.4) and between standard and identical image presentation (*t_24_ =* 3.35, *p* = 0.003, Cohen's *d* = 0.7). These results indicate that swapped binocular image presentation led to an impairment in segmentation performance while also, surprisingly, showing a slight preference for monocular presentation. This was not the case, however, for testing with the monocularly trained U-net model, where dʹ values averaged only 3.34. U-net performance contrasts markedly with the d’ values obtained when our binocular model was presented with identical images. Differences were again significant on a two-tailed, related-samples t-test (*t*_24_ = 19.41, *p* < 0.001, Cohen's *d* = 3.9). This result suggests that binocular presentation during training is beneficial for the learning of monocular segmentation signals.

**Figure 6.**
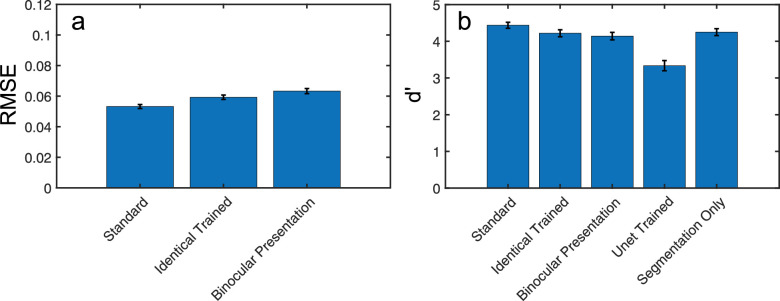
(a) Depth errors (RMSE) for the standard model, plus a version of the model trained and tested on identical image pairs, and one trained on identical image pairs and tested on standard binocular pairs. (b) Segmentation performance plotted as dʹ scores for the same sets of models as in (a), plus the monocular U-net model and a version of the network trained on the segmentation task only. *Error bars* show standard errors on the mean.

In addition to comparing our model's performance against an equivalent, purely monocular, model, we also conducted a further comparison, where our binocular model was trained on identical images and tested either against identical or against standard binocular images. Depth estimation performance for this identical image trained model is shown, compared to our standard model performance in Figure 6a. Depth estimation errors increased for the identical image trained model, compared to our standard model, with RMSE values rising from 0.053 to 0.059 under identical image testing and 0.063 under binocular image testing. These differences were significant on related-samples *t-*tests (*t_99_* = 3.14, *p* = 0.002, Cohen's *d* = 0.3; *t_99_* = 4.77, *p* < 0.001, Cohen's *d* = 0.5). The difference in depth estimation performance for the identical image trained network was also significant for identical, compared with binocular image testing (*t_99_* = 3.66, *p* < 0.001, Cohen's *d* = 0.4). Note that depth estimation errors were still much lower than for identical image and pseudoscopic viewing in the standard model (see Figure 5a), indicating a dependence on pictorial depth cues in the identical image trained model and further demonstrating the impact of binocular depth cues in our standard network.

Segmentation performance for the identical image trained network is shown under both identical image and binocular viewing conditions in Figure 6b. When tested with identical images, this network showed impaired segmentation performance compared to our standard binocular image trained network, with mean object d’ values declining from 4.44 to 4.22 (*t_24_* = 5.38, *p* < 0.001, Cohen's *d* = 1.1), although these performance levels were significantly greater than found with the monocular U-net (*t_24_* = 11.42, *p* < 0.001, Cohen's *d* = 2.3). Segmentation performance for our standard model was also better than for the identical image trained model, tested under binocular viewing conditions with mean d’ values for the latter falling to 4.14 (*t_24_* = 5.52, *p* < 0.001, Cohen's *d* = 1.1). Unlike depth estimation, there was no significant difference in dʹ values for the identical image trained model under these two viewing conditions (*t_24_* = 1.80, *p* = 0.085, Cohen's *d* = 0.4).

The improved performance of our standard model compared to the identical image trained model provides further support for the benefits of binocular image viewing in both depth estimation and scene segmentation. Yet, the improved segmentation scores for this identical image trained model, relative to the monocular U-net model shows that binocular viewing is not the only driver of performance in our network. While one possibility is that these improvements were due to factors such as binocular summation (Baker et al., 2018; Blake & Fox, 1973; Blake et al., 1981), this is not consistent with the lack of impairment in performance when the identical image trained network was tested with standard binocular image pairs. To further examine this issue, we investigated a remaining difference between the monocular U-net model and our binocular network, the presence of a simultaneous depth estimation pathway. We compared the performance of our standard binocular network to a network trained on the segmentation task only (see Figure 6b). Although this binocular trained segmentation network performed substantially better than the monocular U-net, with average dʹ values of 4.25, compared to 3.34 (*t_24_* = 10.54, *p* < 0.001, Cohen's *d* = 2.1 on a related samples *t*-test), this was still lower than average dʹ values found for our standard network (*t_24_* = 5.49, *p* < 0.001, Cohen's *d* = 1.1). These results suggest that simultaneous training on depth estimation and segmentation is itself beneficial for segmentation performance.

### Segmentation and depth estimation

**Figure 7.**
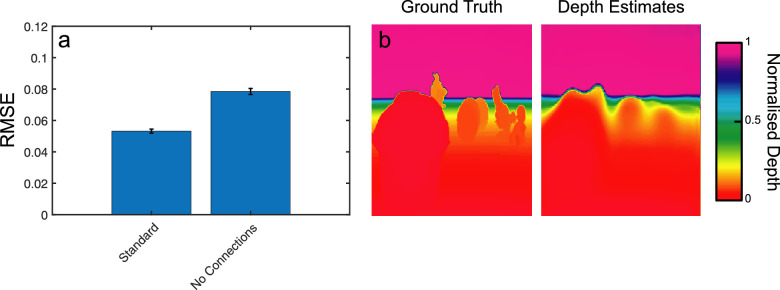
(a) Depth errors (RMSE) for the standard binocular model, as well as a version of the model that removed lateral connections between the network's segmentation pathways and the depth estimation branch. Error bars show standard errors on the mean. (b) An example depth estimation output from the “no lateral connections” model, with accompanying ground truth. Depth estimates were visibly smoothed over space compared with those from the standard binocular model (see Figure 3c).

The preceding comparisons of depth estimation and segmentation performance show clear benefits of binocular presentation in both the training and testing of our network. The architecture of our network, with its lateral connections providing inputs from the segmentation pathways into the depth estimation pathway, also allowed for a further comparison, examining the potential role of segmentation information *for* depth estimation. To examine this relationship, we trained and tested our network under an additional condition where these lateral connections were removed. This left the segmentation pathways unchanged but led to significant impairment of depth estimation performance (Figure 7a). RMSE values across images rose from 0.053 with lateral connections, to 0.078 without (*t_99_* = 10.73, *p* < 0.001, Cohen's *d* = 1.1). Notably, depth estimates from the model without lateral connections varied more smoothly over space, with an absence of sharp depth edges between objects (see Figure 7b).

## Discussion

In this article, we have reported the results of a new DNN model for simultaneous depth estimation and scene segmentation, using binocular image inputs. This model shows selectivity for binocular eye-of-origin signals, a necessary prerequisite for the encoding of both binocular disparities and monocular occlusions. Here, we consider what may be learned about the function of biological visual systems from the performance of this model.

### Binocular image training enhances monocular segmentation

There has been significant debate among researchers in biological vision as to the adaptive significance of binocular vision for real world tasks (cf. Heesy, 2009). Although benefits of binocular viewing have been demonstrated in laboratory situations for a number of tasks, including color constancy (Yang & Shevell, 2002), object tracking and visual search (Dunser & Mancero, 2009; Nakayama & Silverman, 1986; Vishwanathan & Mingolla, 2002), and the perception of shape, depth, distance and heading direction (Brenner & van Damme, 1999; McCann, Hayhoe, & Geisler, 2018; Macuga, Loomis, Beall, & Kelly, 2006; Scarfe & Hibbard, 2006; van den Berg & Brenner, 1994), a number of factors suggest that its benefits may be more limited in natural environments. One commonly raised issue is that deficits in binocular vision are relatively commonplace. Coutant and Westheimer (1993) found that 2.7% of a student population sample had stereoacuity thresholds of greater than 2 arcmin, with 20% unable to meet a stricter stereoacuity criterion of 30 arcsec. Other studies have shown dominance for non-binocular visual cues under multi-cue presentation conditions (Allison & Howard, 2000a; Allison & Howard, 2000b; Hill & Johnston, 2007; van Ee, van Dam, & Erkelens, 2002), including in scene recognition tasks (Valsecchi, Caziot, Backus, & Gegenfurtner, 2013).

Here, we provide evidence for the importance of binocular signals for depth estimation and scene segmentation tasks, while also supporting the idea that, for segmentation at least, we typically depend upon the information from monocular signals. For depth estimation, binocular image presentation resulted in lower average errors than either monocular presentation or pseudoscopic presentation. These results clearly show the benefits of adding correctly arranged binocular signals for estimating depth. In addition, however, they also show that the reversal of these binocular signals, although effective in impairing performance, does not entirely override the information provided by monocular depth cues. In this respect our model behaves in a manner consistent with human observers, where pseudoscopic viewing typically only fully reverses depth in abstract stimuli (van den Enden & Spekreijse, 1989). It should be noted, however, that pseudoscopic perception is not particularly well understood with regards to how binocular and pictorial depth cues interact to determine perceived three-dimensional structure. Our model may therefore prove useful in allowing for the generation of predictions on perceived depth and shape under these viewing conditions.

This benefit for binocular presentation extends to scene segmentation tasks. Model segmentation performance for standard binocular image presentation surpassed that found for pseudoscopically presented images. This effect of presentation order shows that the binocular signals encoded by our network are eye-of-origin dependent, a prerequisite for any subsequent measurement of binocular disparity or monocular occlusion. Yet our results are not simply an indication that binocular information is, by default, critical or beneficial for this task. Two main results complicate this issue. First, identical image presentation offers a small, but significant, benefit over binocular presentation for our model. Second, this benefit for images without binocular disparity information does not extend to a purely monocular version of the segmentation model or to a model trained on identical image pairs.

To understand these issues, one must consider the nature of both monocular and binocular object boundary signals. For binocular cues, although monocular occlusions are effective for signaling object boundaries, they also give rise to the problem of ascribing both depth and object identity to monocular regions. These difficulties could underpin the slightly poorer performance of our model under binocular, compared with identical image, presentation.

A consideration of monocular boundary cues can help to explain the differences in performance between our model, under identical image conditions, and the equivalent, purely monocular, U-net segmentation model. The primary difficulty faced by monocular segmentation processes, is the requirement to differentiate between pattern edges and object edges. As noted, binocular presentation offers a powerful means to resolve this problem through the addition of binocular disparity-defined edges and monocular occlusions. The benefits of these binocular signals are, potentially, twofold. First, they can directly contribute to boundary detection, as under standard binocular viewing conditions. They may, however, also offer systems a means to learn how to distinguish between monocular signals associated with boundary edges and pattern edges. In this way, binocular signals may hold adaptive value not just as segmentation signals in and of themselves, but as a means to support the development of such abilities using other image cues. This finding offers potential insight into developmentally acquired perceptual impairments, such as amblyopia, where disruptions in the development of binocular vision lead to perceptual deficits in both amblyopic and fellow eyes (Parker, Smith, & Krug, 2016; Simmers & Bex, 2004; Simmers, Ledgeway, Hess, & McGraw, 2003). Relatedly, impairments in scene segmentation abilities have also been reported in human patients recovering from early blindness, with signals from motion-defined boundaries seemingly critical for the development of these abilities (Ostrovsky, Meyers, Ganesh, Mathur, & Sinha, 2009).

### Learning depth estimation supports enhanced segmentation

By manipulating the arrangement of binocular image pairs in the training and testing of our network, we have shown that the binocular viewing of scenes enhances segmentation performance and supports the further development of monocular segmentation capabilities. Binocular viewing offers these enhancements whether compared to a purely monocular model, or to a model with the same binocular architecture, trained on identical image pairs. Although our monocular comparison model shows poorer segmentation performance than the identical image trained model, further comparison with a version of our binocular model trained only on the segmentation task suggests that this may be due to a beneficial effect of our dual task training. This suggests that training on both depth estimation and scene segmentation leads to depth-related differences, through the feedback of depth errors into the initial left-right and binocular encoding layers, with these differences acting as beneficial cues for segmentation. This raises the intriguing possibility that information relevant for the scene segmentation task is learned by the network only by virtue of its initial relevance for another task.

### Scene segmentation in the brain

Although any attempts to compare DNN architectures and model weights to human and nonhuman animal physiology should be made with extreme caution (Lopez-Rubio, 2018; Majaj & Pelli, 2018), it is worth considering where similarities do exist and how this may inform our knowledge of biological visual systems. Critically, we do not view any similarities between the structure of our network and mammalian physiology as essential for our findings to hold relevance for our understanding of biological visual systems. Although the learning that occurred within our network need not necessarily translate to the information learned by biological systems, the benefits of binocular viewing for segmentation and depth estimation in our network may still be considered as hypotheses on the likely pattern of sensitivities to be found in such biological systems. The model reported here offers the possibility for two comparisons to biological vision. First, the architecture of our network maps onto several known aspects of the physiology of mammalian visual systems. As in such biological systems, our network begins with monocular encoding stages, followed by a point of binocular convergence. After this point the network proceeds along specialized pathways for segmentation and depth estimation.

This specialization is similar, in some respects, to the divergence of dorsal and ventral pathways in humans (cf., de Hann & Cowey, 2011). Although binocular processing occurs in multiple areas of the human brain, it does so in very different ways with, for example, ventral areas V4, TE and IT showing selectivity for both surface segmentation and descriptors of surface shape (Verhoef, Vogels, & Janssen, 2016). That our model is able to simultaneously learn and perform both segmentation and depth estimation tasks shows the suitability of our common encoding pathway as a generalized stage of visual processing. As noted in section 4.2, there may be additional benefits to these common early processing stages, where selectivity for signals may develop in response to specific task requirements but may also then prove useful for a range of other tasks.

A second point of comparison between our network and human vision comes in the use of connections between our specialized segmentation and depth estimation output pathways. These connections enhance depth estimation performance, with RMSE values for depth estimation increasing significantly in their absence. Depth estimates without lateral connections were also notably poorer across object boundaries, with a visible smoothing of depth between objects. Not only do these lateral connections map onto those found between functionally specialized areas in the mammalian brain (Cloutman, 2013; de Haan & Cowey, 2011; Schenk & McIntosh, 2010), their measured effects also provide a qualitative match to the psychophysical behavior of human observers in a number of depth perception tasks (Cammack & Harris, 2016; Deas & Wilcox, 2014; Deas & Wilcox, 2015; Goutcher et al., 2018; Goutcher & Wilcox, 2021; Mamassian & Zannoli, 2020; Wardle & Gillam, 2016). This psychophysical work has demonstrated the importance of segmentation boundaries on perceived depth, with Mamassian and Zannoli (2020) suggesting that object boundaries are used to delimit depth averaging processes. The benefits of lateral connections between segmentation and depth estimation pathways in our network support this suggestion.

## Conclusions

The novel DNN reported in this article shows high levels of accuracy in scene segmentation and depth estimation tasks and is capable of learning and performing these dual tasks simultaneously. By manipulating the arrangement of binocular images, we have shown that this model encodes eye-of-origin information, necessary for the encoding of both binocular disparities and monocular half-occlusions. Results from these manipulations show that binocular signals are informative for both tasks and may play a role in supporting the learning of monocular segmentation signals. They further show that the simultaneous learning of depth estimation and segmentation tasks may in itself be beneficial for the development of sensitivity to depth-related segmentation cues. These findings provide a further example to illustrate the usefulness of deep learning approaches as a tool for understanding biological vision, providing evidence on where and how stimulus information can be used and recovered by suitable network structures.

## Supplementary Material

Supplement 1
