# Supplementary material for: Binocular vision supports the development of scene segmentation capabilities: Evidence from a deep learning model
Source: J Vis. 2021 Jul 21;21(7):13. doi: 10.1167/jov.21.7.13 (PMC8300045; doi:10.1167/jov.21.7.13)
Supplement: Supplement 1 [file jovi-21-7-13_s001.docx]

**Supplementary Table 1.**

| **Input Layer** | | | |
| --- | --- | --- | --- |
| **L0/R0** | **Layer Type** | **Layer Specification** | **Activation Function** |
| Input | Input | 224 x 224 x 3 | n/a |

| **Left/Right Image Encoding Pathways** | | | |
| --- | --- | --- | --- |
| **L1/R1** | **Layer Type** | **Layer Specification** | **Activation Function** |
| conv_1 | Convolution | Filters = 16, stride = (3,3), padding = same | ReLu |
| norm_1 | Batch Normalisation | n/a | n/a |
| conv_2 | Convolution | Filters = 16, stride = (3,3), padding = same | ReLu |
| norm_2 | Batch Normalisation | n/a | n/a |
| pool_1 | Max Pooling 2D | Pool size = (2,2), padding = same, output size = 112 x 112 | n/a |
| conv_3 | Convolution | Filters = 32, stride = (3,3), padding = same | ReLu |
| norm_3 | Batch Normalisation | n/a | n/a |
| conv_4 | Convolution | Filters = 32, stride = (3,3), padding = same | ReLu |
| norm_4 | Batch Normalisation | n/a | n/a |
| pool_2 | Max Pooling 2D | Pool size = (2,2), padding = same, output size = 56 x 56 | n/a |
| conv_5 | Convolution | Filters = 64, stride = (3,3), padding = same | ReLu |
| norm_5 | Batch Normalisation | n/a | n/a |
| conv_6 | Convolution | Filters = 64, stride = (3,3), padding = same | ReLu |
| norm_6 | Batch Normalisation | n/a | n/a |
| pool_3 | Max Pooling 2D | Pool size = (2,2), padding = same, output size = 28 x 28 | n/a |
| conv_7 | Convolution | Filters = 128, stride = (3,3), padding = same | ReLu |
| norm_7 | Batch Normalisation | n/a | n/a |
| conv_8 | Convolution | Filters = 128, stride = (3,3), padding = same | ReLu |
| norm_8 | Batch Normalisation | n/a | n/a |
| pool_4 | Max Pooling 2D | Pool size = (2,2), padding = same, output size = 14 x 14 | n/a |
| conv_9 | Convolution | Filters = 256, stride = (3,3), padding = same | ReLu |
| norm_9 | Batch Normalisation | n/a | n/a |
| conv_10 | Convolution | Filters = 256, stride = (3,3), padding = same | ReLu |
| norm_10 | Batch Normalisation | n/a | n/a |

| **Binocular Combination Layer** | | | |
| --- | --- | --- | --- |
| **C1** | **Layer Type** | **Layer Specification** | **Activation Function** |
| Left_right_concat | Concatenation | 14 x 14 x 512 | n/a |

| **Left/Right Image Segmentation Pathways** | | | |
| --- | --- | --- | --- |
| **L2/R2** | **Layer Type** | **Layer Specification** | **Activation Function** |
| conv_11 | Convolution | Inputs = C1 (14 x 14 x 512) , Filters = 256, stride = (3,3), padding = same | ReLu |
| norm_11 | Batch Normalisation | Filters = 256, stride = (3,3), padding = same | n/a |
| conv_12 | Convolution | Filters = 256, stride = (3,3), padding = same | ReLu |
| norm_12 | Batch Normalisation | n/a | n/a |
| concat_1 | Concatenation | Upsampling2D(left_norm_12), size=(2,2)/left_norm_8 | n/a |
| conv_13 | Convolution | Filters = 128, stride = (3,3), padding = same | ReLu |
| norm_13 | Batch Normalisation | n/a | n/a |
| conv_14 | Convolution | Filters = 128, stride = (3,3), padding = same | ReLu |
| norm_14 | Batch Normalisation | n/a | n/a |
| concat_2 | Concatenation | Upsampling2D(left_norm_14), size = (2,2)/left_norm_6 | n/a |
| conv_15 | Convolution | Filters = 64, stride = (3,3), padding = same | ReLu |
| norm_15 | Batch Normalisation | n/a | n/a |
| conv16 | Convolution | Filters = 64, stride = (3,3), padding = same | ReLu |
| norm_16 | Batch Normalisation | n/a | n/a |
| concat_3 | Concatenation | Upsampling2D(left_norm_16), size = (2,2)/left_norm_4 | n/a |
| conv_17 | Convolution | Filters = 32, stride = (3,3), padding = same | ReLu |
| norm_17 | Batch Normalisation | n/a | n/a |
| conv_18 | Convolution | Filters = 32, stride = (3,3), padding = same | ReLu |
| norm_18 | Batch Normalisation | n/a | n/a |
| concat_4 | Concatenation | Upsampling2D(left_norm_18),size = (2,2)/left_norm_2 | n/a |
| conv_19 | Convolution | Filters = 16, stride = (3,3), padding = same | ReLu |
| norm_19 | Batch Normalisation | n/a | n/a |
| conv_20 | Convolution | Filters = 16, stride = (3,3), padding = same | ReLu |
| norm_20 | Batch Normalisation | n/a | n/a |

| **Left/Right Segmentation Decision Layer** | | | |
| --- | --- | --- | --- |
| **L3/R3** | **Layer Type** | **Layer Specification** | **Activation Function** |
| Left_output/ right_output | Convolution | Filters = n_classes, stride = (1,1), padding = same | Softmax |

| **Depth Estimation Pathway** | | | |
| --- | --- | --- | --- |
| **D1** | **Layer Type** | **Layer Specification** | **Activation Function** |
| Depth_conv_1 | Convolution | Input = C1 ( 14 x 14 x 512),  Filters = 16, stride = (3, 3), padding = same, output size = 14 x 14 x 16 | ReLu |
| Depth_norm_1 | Batch Normalisation | n/a | n/a |
| Depth_conv_2 | Convolution | Filters = 16, stride = (3,3), padding = same | ReLu |
| Depth_norm_2 | Batch Normalisation | n/a | n/a |
| Depth_conv_3 | Convolution | Filters = 32, stride = (3,3), padding = same | ReLu |
| Depth_norm_3 | Batch Normalisation | n/a | n/a |
| Depth_conv_4 | Convolution | Filters = 32, stride = (3,3), padding = same | ReLu |
| Depth_norm_4 | Batch Normalisation | n/a | n/a |
| Depth_conv_5 | Convolution | Filters = 64, stride = (3,3), padding = same | ReLu |
| Depth_norm_5 | Batch Normalisation | n/a | n/a |
| Depth_conv_6 | Convolution | Filters = 64, stride = (3,3), padding = same | ReLu |
| Depth_norm_6 | Batch Normalisation | n/a | n/a |
| Depth_conv_7 | Convolution | Filters = 128, stride = (3,3), padding = same | ReLu |
| Depth_norm_7 | Batch Normalisation | n/a | n/a |
| Depth_conv_8 | Convolution | Filters = 128, stride = (3,3), padding = same | ReLu |
| Depth_norm_8 | Batch Normalisation | n/a | n/a |
| Depth_conv_9 | Convolution | Filters = 256, stride = (3,3), padding = same | ReLu |
| Depth_norm_9 | Batch Normalisation | n/a | n/a |
| Depth_conv_10 | Convolution | Filters = 256, stride = (3,3), padding = same | ReLu |
| Depth_norm_10 | Batch Normalisation | n/a | n/a |
| Depth_conv_11 | Convolution | Filters = 256, stride = (3,3), padding = same | ReLu |
| Depth_norm_11 | Batch Normalisation | n/a | n/a |
| Depth_conv_12 | Convolution | Filters = 256, stride = (3,3), padding = same | ReLu |
| Depth_norm_12 | Batch Normalisation | n/a | n/a |
| **D2** | **Layer Type** | **Layer Specification** | **Activation Function** |
| Depth_conv_13 | Convolution | Filters = 128, stride = (3,3), padding = same | ReLu |
| Depth_norm_13 | Batch Normalisation | n/a | n/a |
| Depth_conv_14 | Convolution | Filters = 128, stride = (3,3), padding = same | ReLu |
| Depth_norm_14 | Batch Normalisation | n/a | n/a |
| Depth_pool_1 | Max Pooling | Pool size = (2,2), padding = same, output size = 7 x7 x 128 | n/a |
| Depth_concat_1 | Concatenation | Inputs = Upsampling2D(Depth_pool_1), size = (2,2)/left_norm_10/ right_norm_10 | n/a |
| Depth_conv_15 | Convolution | Filters = 64, stride = (3,3), padding = same | ReLu |
| Depth_norm_15 | Batch Normalisation | n/a | n/a |
| Depth_conv_16 | Convolution | Filters = 64, stride = (3,3), padding = same | ReLu |
| Depth_norm_16 | Batch Normalisation | n/a | n/a |
| Depth_concat_2 | Concatenation | Inputs = Upsampling2D(Depth_norm16), size = (2,2)/left_norm_8/ right_norm_8 | n/a |
| Depth_conv_17 | Convolution | Filters = 32, stride = (3,3), padding = same | ReLu |
| Depth_norm_17 | Batch Normalisation | n/a | n/a |
| Depth_conv_18 | Convolution | Filters = 32, stride = (3,3), padding = same | ReLu |
| Depth_norm_18 | Batch Normalisation | n/a | n/a |
| Depth_concat_3 | Concatenation | Inputs = Upsampling2D(Depth_norm_18), size = (2,2)/ left_norm_6/ right_norm_6 | n/a |
| Depth_conv_19 | Convolution | Filters = 16, stride = (3,3), padding = same | ReLu |
| Depth_norm_19 | Batch Normalisation | n/a | n/a |
| Depth_conv_20 | Convolution | Filters = 16, stride = (3,3), padding = same | ReLu |
| Depth_norm_20 | Batch Normalisation | n/a | n/a |
| Depth_up_1 | Upsampling2D | Size = (2,2) | n/a |
| Depth_conv_21 | Convolution | Filters = 16, stride = (3,3), padding = same | ReLu |
| Depth_norm_21 | Batch Normalisation | n/a | n/a |
| Depth_conv_22 | Convolution | Filters = 16, stride = (3,3), padding = same | ReLu |
| Depth_norm_22 | Batch Normalisation | n/a | n/a |
| Depth_up_2 | Upsampling2D | Size = (2,2) | n/a |

| **Depth Estimation Decision Layer** | | | |
| --- | --- | --- | --- |
| **D3** | **Layer Type** | **Layer Specification** | **Activation Function** |
| Depth_output | Convolution | Filters = 1, stride = (1,1), padding = same | Linear |

*Supplementary Table 1. Details for all network layer computations. Layer codes (L1, L2, etc) refer to network stages shown in Figure 2*
